# Extracellular Regulation of Sperm Transmembrane Adenylyl Cyclase by a Forward Motility Stimulating Protein

**DOI:** 10.1371/journal.pone.0110669

**Published:** 2014-10-28

**Authors:** Souvik Dey, Debarun Roy, Gopal C. Majumder, Debdas Bhattacharyya

**Affiliations:** Division of Cryobiology, Centre for Rural and Cryogenic Technologies, Jadavpur University, Kolkata, West Bengal, India; Universidad Nacional Autónoma de México, Mexico

## Abstract

Forward motility stimulating factor (FMSF), a glycoprotein isolated from buffalo serum, binds to the surface of the mature sperm cells to promote their progressive motility. This article reports the mode of signal transduction of this extracellular factor in goat sperm. The mechanism was investigated by assaying intracellular second messenger level and forward motility in presence of different pharmacological modulators. Mg^++^-dependent Forskolin responsive form of transmembrane adenylyl cyclase (tmAC) of goat spermatozoa was probed for its involvement in FMSF action. Dideoxyadenosine, a selective inhibitor of tmACs, was used to identify the role of this enzyme in the scheme of FMSF-signaling. Involvement of the α-subunit of G-protein in this regard has been inspected using GTP_γ_S. Participation of protein kinase A (PKA) and tyrosine kinase was checked using IP20 and genistein, respectively. FMSF promotes tmAC activity in a dose-dependent manner through receptor/G-protein activation to enhance intracellular cAMP and forward motility. Motility boosting effects of this glycoprotein are almost lost in presence of dideoxyadenosine. But, FMSF displayed substantial motility promoting activity when movement of spermatozoa was inhibited with KH7, the specific inhibitor of soluble adenylyl cyclase indicating tmAC to be the primary target of FMSF action. Involvement of cAMP in mediating FMSF action was confirmed by the application of dibutyryl cAMP. Observed motility regulatory effects with IP20 and genistein indicate contribution of PKA and tyrosine kinase in FMSF activity; enhanced phosphorylation of a tyrosine containing ≈50 kDa protein was detected in this regard. FMSF initiates a novel signaling cascade to stimulate tmAC activity that augments intracellular cAMP, which through downstream crosstalk of phosphokinases leads to enhanced forward motility in mature spermatozoa. Thus, this article for the first time describes conventional tmAC-dependent profound activation of progressive motility by a physiologic extracellular factor in a mammalian species.

## Introduction

After ejaculation into female reproductive tract, spermatozoa travel a considerable distance to contact the mature ovum and fertilize it. Apart from acquisition of other physiologic competence [1] the sperm cells are required to be progressively motile to reach their goal [2]. This characteristic of forward motility of male gamete cells is primarily achieved during the epididymal transit [3]; however the cells attain the ultimate kinetic potential inside the female genital tract [4]. Reports of various types of extracellular sperm motility promoting factors in male reproductive fluids and blood serum of different animals, as well as human, have been part of the scientific literature for the last four decades [2], [5], [6], [7], [8], [9], [10]. But, none of these motility-regulatory entities were sufficiently purified and characterized. In the last few years, some progress has been made in this regard [11], [12], [13], [14], [15], [16]. Forward motility stimulating factor (FMSF) is a 66 kDa, Mg^++^-dependent glycoprotein (pI ≈ 3.7) isolated from buffalo serum; it has been shown to significantly and selectively promote progressive motility at sub-micromolar doses in caprine (*Capra hircus*) caudal mature spermatozoa without having any role in cell anti-sticking activity [17], [11]. This patented biomolecule is partially heat-stable and spherical in shape [18], [19], [11]; it is glycosylated with mannose residue, which is essential for its activity. Previous observations have suggested that this extracellular factor has no species specificity in its activity on sperm cells; its efficacy in augmenting forward motility of spermatozoa of other mammalian species beside buffalo is also evident. This stimulatory glycoprotein is a part of the physiologic milieu of uterine fluid of at least two cattle species (viz. buffalo and bull) and goat [16], where it is present in the epididymal plasma too. For that reason, FMSF has been implicated to have potential role in cattle breeding protocols. Recent findings show FMSF to be present throughout the length of goat epididymal fluid, especially in the caudal segment where its concentration nearly is 300 nM [20]. The receptor of this factor appears on the sperm cell surfaces at the terminal maturational phase of these cells [16]. Binding of FMSF with its cognate receptor on sperm cell surface has been shown to be very specific. Unpublished observation suggests this *in vitro* interaction to be almost irreversible under moderately dissociating condition. The identification of FMSF receptor has brought new dimension to the way sperm cell maturation can be explained. Attainment of progressive movement of the germ cells has been found to be linked with the expression of this receptor.

However, little is known regarding the mechanism of action of these extracellular motility stimulating proteins with special reference to their intrasperm signaling patterns. This investigation reports a motility-stimulating protein dependent cell signaling mechanism using the purified serum FMSF and goat sperm as the model system. During the present study, evidence was found which demonstrates for the first time that this extracellular protein exhibits its stimulating potential by activating the transmembrane adenylyl cyclase which plays a pivotal role in the regulation of sperm forward motility.

## Materials and Methods

CM-cellulose, DEAE-cellulose, Sephadex G-100, Concanavalin A-sepharose 4B, Iodoacetamide, KH7, cAMP-dependent protein kinase inhibitor (IP20), db-cAMP, GTPγS, GDP, dialysis membrane (MWCO 10 kDa), dextran (MW 228 kDa), forskolin, phenyl methylsulfonyl fluoride, protease inhibitor cocktail, penicillin and cAMP Enzyme Immunoassay Kit, Direct (CA200) were obtained from Sigma Chemical Company (St. Louis, MO). 2,3-Dideoxyadenosine (ddAdo) was purchased from Santa Cruz Biotechnology, USA. Amicon ultra centrifugal device (MWCO 10 kDa) was obtained from Millipore Corporation (Billerica, MA). Ammonium sulfate, Brilliant blue G-250, orthophosphoric acid, Ammonium bicarbonate, TCEP, acetonitrile, β-mercaptoethanol, bovine serum albumin, EDTA sodium salt, nickel chloride, polyethyleneglycol (MW 15–20 kDa), sodium deoxycholate, sodium dodecyl sulfate, sodium fluoride and phosphocreatinine were procured from Sisco Research laboratories, Mumbai, India. Protein molecular weight marker was purchased from Fermentus Life Sciences. Super Signal West Pico chemiluminiscent substrate (Cat no. 34087) was purchased from Thermo Scientific. Cell signaling make anti-phoshothreonine (Cat no. 9836) and anti-phoshotyrosine antibodies (Cat no. 9411) were gifted by Dr. M.K. Kundu, Bose Institute, Kolkata. Amersham make protein A-FITC conjugate and Pierce Trypsin Protease, MS Grade was presented by Dr. S. Das and Mr. R Majumder, IICB, Kolkata, respectively.

### Ethics Statement

No live animals were used in any of the experiments described in this article. Buffalo blood and epididymal tissue of goat (*Capra hircus*) are slaughterhouse waste. These tissues were collected from government regulated (with prior permission from Department of Health, Kolkata Municipal Corporation, ref. letter dated 23.05.11)/local slaughterhouses within an hour of sacrifice of the animal and after use, disposed according to the norms of State Pollution Control Board to avoid possible biohazard.

### Mass Spectral (MS) analysis of FMSF derived peptides

FMSF was purified from buffalo serum according to the method described earlier by Mandal et al., (2006) [11] with necessary modifications [16]. In short, it was serially purified through precipitation with 60–80% ammonium sulfate, elution through CM-cellulose ion exchange resin with 0.2 mM NaCl in 10 mM Na-acetate buffer of pH 5.6, molecular sieving by Sephadex G-100 gel filtration column (retention time 63 min), elution from non-denaturing 7.5% PAGE (R_f_ value 0.6) and finally by eluting with 100 mM β-methyl mannoside in 20 mM Tris-Cl/0.5 M NaCl buffer, pH 7.4 from concanavalin A column. Variability found with activity of different purified batches of the glycoprotein was found to be <10%. Purified protein was run into 10% SDS-PAGE, stained with colloidal Coomassie solution and excised using a fine scalpel. After destaining, it was reduced with 50 mM TCEP solution at 60°C for 10 min. After cooling and removal of the reducing reagent, it was alkylated with 100 mM iodoacetamide solution in the dark at room temperature (RT ≈ 27–28°C) for 1 hr. Iodoacetamide solution was removed, and washed the gel pieces twice with 50% (v/v) acetonitrile (ACN) and 25 mM ammonium bicarbonate solution. Then gel pieces were shrinked with sufficient ACN. Gel pieces were air dried after removal of ACN and rehydrated with 100 ng/µl trypsin solution to just cover the gel piece, and incubated at RT for 15 min. Digestion buffer (25 mM NH_4_HCO_3_ solution) was added and incubated over night at RT. Tubes were then centrifuged to obtain digested peptide solution devoid of gel pieces. The supernatant extract was desalted before its application to the mass spectrometry analysis. From the final solution, 0.5 µl was spotted on an α-Cyano-4-hydroxy-cinnamic acid matrix. Analysis of the sample was done by an Applied Biosystems 4800 MALDI TOF/TOF Analyzer. Sequences of peptides were identified using Expasy proteomics and sequence analysis tool.

### Isolation of caprine epididymal spermatozoa and epididymal plasma (EP)

Spermatozoa were obtained from goat cauda-epididymides by the procedure described earlier [11]. Spermatozoa were extracted at room temperature from the epididymides in a modified Ringer’s solution (RPS) free of Ca^2+^ (Medium B: 119 mM NaCl, 5 mM KCl, 1.2 mM MgSO_4_, 10 mM glucose, 16.3 mM potassium phosphate buffer, pH 7^.^5; penicillin, 50 U/ml) [13]. The sperm preparations were used immediately (within 15 min) for motility assays. Goat cauda EP was prepared by the procedure described earlier [21] with suitable modifications. Freshly extracted sperm preparation (minimum 1×10^8^ cells/ml) was first centrifuged at 800 g for 10 min when most of the spermatozoa were removed as pellet. The resulting supernatant, which was slightly turbid, was spun again at 14,000 g for 30 min to obtain cell free EP in the supernatant fraction. EP contains the anti-sticking factor (ASF) that prevents adhesion of washed spermatozoa with glass surface of hemocytometer during motility assays. This helps in determination of true forward motility of the treated cells without any artifact [21]. The concentrations of EP in the assays were expressed as its protein content.

### Cell viability assay

Viability of the sperm cells at different conditions was measured by the trypan blue dye-exclusion assay according to the protocol of Strober (2001) [22]. In brief, 0.4% trypan blue was used at the ratio of 1∶10 with sperm cell suspension for ≈ 5–7 min incubation period and subsequently cells were counted in hemocytometer; spermatozoa that took no blue coloration were regarded as viable.

### Forward motility assay of FMSF under the influence of different pharmacological modulators

The effect of FMSF on mature caudal spermatozoa to cause stimulation of forward motility was measured by the standard procedure with some modifications [13]. Spermatozoa (0.5×10^6^ cells) were incubated with EP (0.6 mg protein) in absence (ovalbumin used as control) or presence of specified amount of FMSF (0, 0.25, 0.5 & 1 µM for 2 min) or pharmacological modulators like forskolin (0, 10, 25, 50, 100 & 200 µM) - a specific tmAC activator [23], ddAdo (0, 100, 225 & 350 µM), a cell-permeable selective p-site inhibitor of tmAC [24], [25] db-cAMP (0.5 mM) - a cell-permeable synthetic analogue of cAMP [26], KH7 (25 µM) – a specific inhibitor of soluble adenylyl cyclase and genistein (0, 10 & 100 µM) - a cell-permeable tyrosine-kinase inhibitor [27] at room temperature (RT, 32°C) for 1 min in a total volume of 0.5 ml of RPS free of Ca^2+^. Saponin (3 µg/ml) was used to permeabilize the spermatozoa for entry of macromolecules [28] without affecting their mobility; these permeabilized cells were used for assay with IP20 (0, 50 & 100 µM), a competitive peptide inhibitor of cAMP-dependent protein kinase, i.e. PKA [29]. In all the cases FMSF was added post-treatment with chemical modulators. Spermatozoa which showed well defined forward motility (excluding cells that moved in small or large circles) were selected and total cell numbers were counted in hemocytometer under a phase contrast microscope at 100× magnification and rechecked by a collaborator who was unaware of any treatments or its possible implications on the forward motility profile of the sperm cells. The percentage of FM cells was then calculated. Systems lacking FMSF served as the blanks in all assays.

### Spectrophotometric velocity analysis of spermatozoa

Vertical velocity of spermatozoa comes across as a superior yardstick for judging sperm quality [30], [31], [32]. For this experiment, freshly extracted preparation of caudal epididymal spermatozoa from caprine species (minimum 5×10^6^ cells) was mixed at final concentration of 2% ficoll in RPS medium. A control was set without treatment of FMSF. FMSF and ddAdo were added at concentrations of 0.5 µM and 225 µM, respectively. The cell suspensions were carefully layered with a Hamilton syringe at the bottom of a standard cuvette containing RPS medium. Sperm vertical motility was measured spectrophotometrically as an increase of absorbance at 545 nm as the cells moved to light path after crossing the 1.8 mm mask by swimming upwards against gravity [31]. Continuous change in absorbance as a function of time was monitored for 10 min with Thermo Scientific Helios-Zeta spectrophotometer equipped with a digital recorder. Then the contents of the cuvettes were mixed and the absorbance for all the cells was noted as A_total_. The percentage of cells that showed vigorous vertical motility (VMC) to move upward into the light path was then calculated as follows: VMC = (A_t_/A_total_)×100, where A_t_ indicates absorbance at certain time points.

#### Transmembrane adenylyl cyclase (tmAC) assay

Fresh caudal sperm membrane was prepared according to the method described earlier [33], [34], [35], [36]. Purity of the plasma membrane was confirmed by phase contrast and electron microscopic studies and analysis of marker enzymes characteristic of cellular organelles [33]. An optimum condition was developed for the assessment of transmembrane adenylyl cyclase activity of the purified membrane fraction; effect of different concentrations of Mg^++^, F^−^, ATP, PCr, Ni^++^, HCO_3_^−^, Ca^++^, ddAdo, KH7 and forskolin in the assay system were evaluated in this regard. To determine the AC stimulating activity, different doses of FMSF (0, 0.125, 0.25 & 0.4 µM) in the standardized assay buffer containing 50 mM tris-Cl (pH 8.5), 2 mM MgCl_2,_ 0.5 mM NaF (to inhibit non-specific adenosine 5′-triphosphatase), 0.5 mM phosphocreatinine (to decrease the inhibition from ADP, via phosphocreatine kinase) and 1.5 mM ATP-Na_2_ [37] were used to determine the AC stimulating activity. To assess involvement of G-protein, one set was treated with GTPγS (10 µM) and the corresponding buffer contained 0.5 mM GDP in addition to the other constituents [38]. The assay buffer was preincubated for 10 min at 37°C. After addition of FMSF (in presence/absence of GTPγS), incubation was done at 37°C for ≈30 min to properly assess the effect GTPγS on the enzyme activity. Reaction was stopped by placing tubes in boiling water bath for 1 min. Then the tubes were centrifuged and the supernatants were used to detect cAMP concentration as per instruction of cAMP-EIA kit, Sigma.

#### Assessment of intracellular cAMP status

Caprine caudal washed spermatozoa (1×10^5^ in each set) were incubated with different doses of FMSF (0, 0.25, 0.5 & 1 µM; time 2 min) and ddAdo (0, 100, 225 & 350 µM; time 30 min in presence of 0.5 µM FMSF) at RT and the cells were pelleted down. These were lysed with 0.1 M HCl for 40–60 min at RT as per instruction of cAMP-EIA kit, Sigma (CA200). After centrifugation the supernatant containing cAMP was estimated with the above kit.

### Kinase assays

To extract the cytosolic fraction, untreated/FMSF treated sperm cells (5×10^6^) were pelleted down at 800×g. The pellet was dissolved in a hypotonic solution of 1.25 mM EDTA-Na_2_ solution of pH 7.6 containing 0.2 mM PMSF to swell at 4°C for 20 min. It was again centrifuged and dispersed in twice the volume of hypotonic buffer. Then 20–25 strokes of Dounce homogenizer was applied to it on ice. The cells were centrifuged at 18,000×g for 30 min and the supernatant was dialyzed extensively in RPS. Protein was estimated by Lowry’s method [39]. The phosphoprotein kinase assay mixture contained 10 µmol sodium glycerophosphate-HCl buffer (pH 5·5), 2 µmol cobalt chloride, 30 nmol EGTA, 6 nmol ATP, 60 µmol sodium chloride, 2 µmol sodium fluoride, 0·5 mg of casein (as substrate) and 0.2 mg of cytosolic protein extract as source of enzyme in a total volume of 0·2 ml. It was then incubated out at 37°C for 20 min [40] and the reaction was stopped by placing in boiling water for 2 min. The assay mixture was centrifuged and the supernatant was used for standard ELISA test [11] using anti-phosphoserine/threonine (for protein kinase A) and tyrosine (for tyrosine kinase) antibodies; absorbance was taken at 492 nm. A standard curve was considered for calculating protein kinase activity (expressed as nmoles of phosphate/mg of protein/min) from absorbance values.

### Quantification of serine/threonine and tyrosine phosphorylation by flow cytometry

Washed caudal spermatozoa were at first treated with the previously mentioned modulators. A total of 1×10^6^ spermatozoa in each set of cells were first permeabilized with 0.1% TritonX-100 to detect intracellular changes. Then, the cells were incubated separately with anti-phosphoserine/threonine and tyrosine antibodies and subsequently fixed with 4% paraformaldehyde. After further washing, ∼1 µg of protein A-FITC was mixed with each preparation and kept at 4°C for 30 min in dark [15]. Appropriate controls (cells with only protein A-FITC treatment and cells lacking FMSF treatment) were kept. Finally, labeled sperm suspensions were analyzed in a FACSCalibur flow-cytometer (Becton Dickinson, Franklin Lakes, NJ) equipped with argon laser (performed at Chittaranjan National Cancer Institute, Kolkata). The non-sperm specific events (mostly small particles, 2–3% of total events) were gated out. The flow cytometric data were stored and analyzed on Becton Dickinson, CellQuest Pro software.

### Imaging of intracellular phosphorylation sites with indirect immunofluorescence technique

Experiments were designed to locate the intracellular tyrosine phosphorylation sites in caudal cells in the absence/presence of FMSF for 10 min. After treatment, 100–120 spermatozoa were attached on each gelatin coated glass slide and fixed with 4% paraformaldehyde [15]. Then the cells were permeabilized and quenched with 0.1% of TritonX-100 and sodium borohydride, respectively. After blocking with 3% BSA in PBS, different sets of cells were incubated separately with anti-phosphotyrosine antibody. Following further washing with PBS, protein A-FITC conjugate was added at a dilution of 1∶40 and finally the cells were visualized in fluorescence mounting media under Ayrus epi-fluorescence microscope at 600× magnifications.

### Western blotting of tyrosine phosphorylated proteins

For detection of the tyrosine phosphorylated proteins, Western blot procedure was followed. The cells (5×10^6^) were first treated with/without of FMSF for 10 min. The protein fraction was extracted by treating cells with RIPA buffer (50 mM Tris-HCl, 150 mM NaCl, 0.1% NP-40, 1.5% SDS, 0.25% Sodium deoxycholate, 1 mM EDTA, 50 mM NaF, 1 mM PMSF) for 30 min followed by high speed centrifugation. The sperm membrane protein was isolated as described earlier and then treated with the solubilizing buffer. The protein fractions were concentrated, run on 10% SDS-PAGE and transferred to PVDF membrane by Amersham transblot apparatus. Nonspecific binding sites were blocked with 5% skimmed milk plus 2% BSA in TBS. The PVDF paper was then incubated with anti-phosphotyrosine antibody (at 1∶2000 dilution followed by horseradish peroxidase-conjugated anti-mouse secondary IgG (at 1∶2500 dilution). After further washing, immunoreactive bands were visualized using a chemiluminescent substrate and developed on photographic film. The PVDF membrane was then reprobed with anti-GAPDH antibody only for the lanes with whole cell lysate proteins.

### Statistical Analysis

The results were expressed as means ± SEM. The data were statistically analyzed by Microcal Originpro version 8.0 (USA). Significance was tested using one-way ANOVA.

## Results

### Mass spectroscopic analysis of FMSF derived peptides

**Figure 1:**
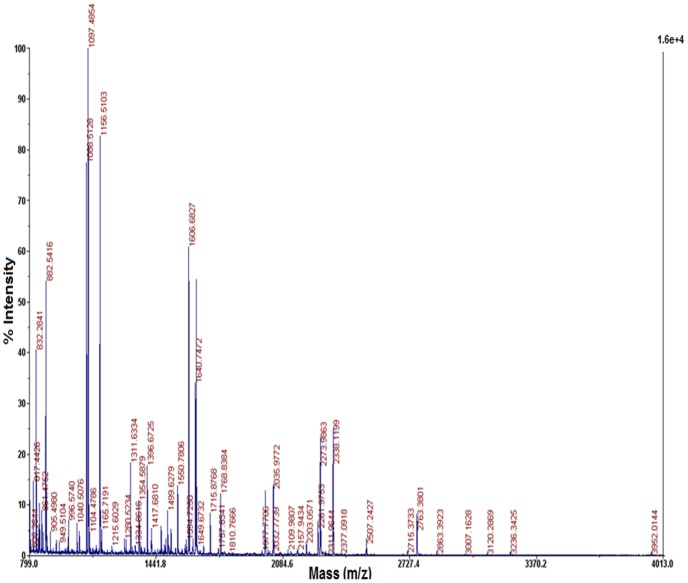
MALDI-TOF spectrum of trypsin digested peptides of FMSF. Three concentrations of the same sample were spotted on the CHCA matrix for analysis, which produced qualitatively identical results. All the peaks on the spectrum were checked for homology in the online protein database; only those with a score >65 were considered significant for discussion in the result section.

**Figure 1** displays the spectrum of trypsin digested FMSF peptides done by MALDI-TOF analysis. The novelty of the protein is evident from the spectral pattern. No similar protein was found to be identified from buffalo serum, till date. Around 26% to 37% sequence homology was found with certain serum glycoproteins (viz. transferrin, accession no. gi 114326282, gi 209973077 and hemiferrin/transferring-like protein, accession no. gi 28849947) suggesting a possible evolutionary relation of FMSF with these proteins.

### Establishment of adenylyl cyclase activity of goat-sperm membrane fraction

**Figure 2:**
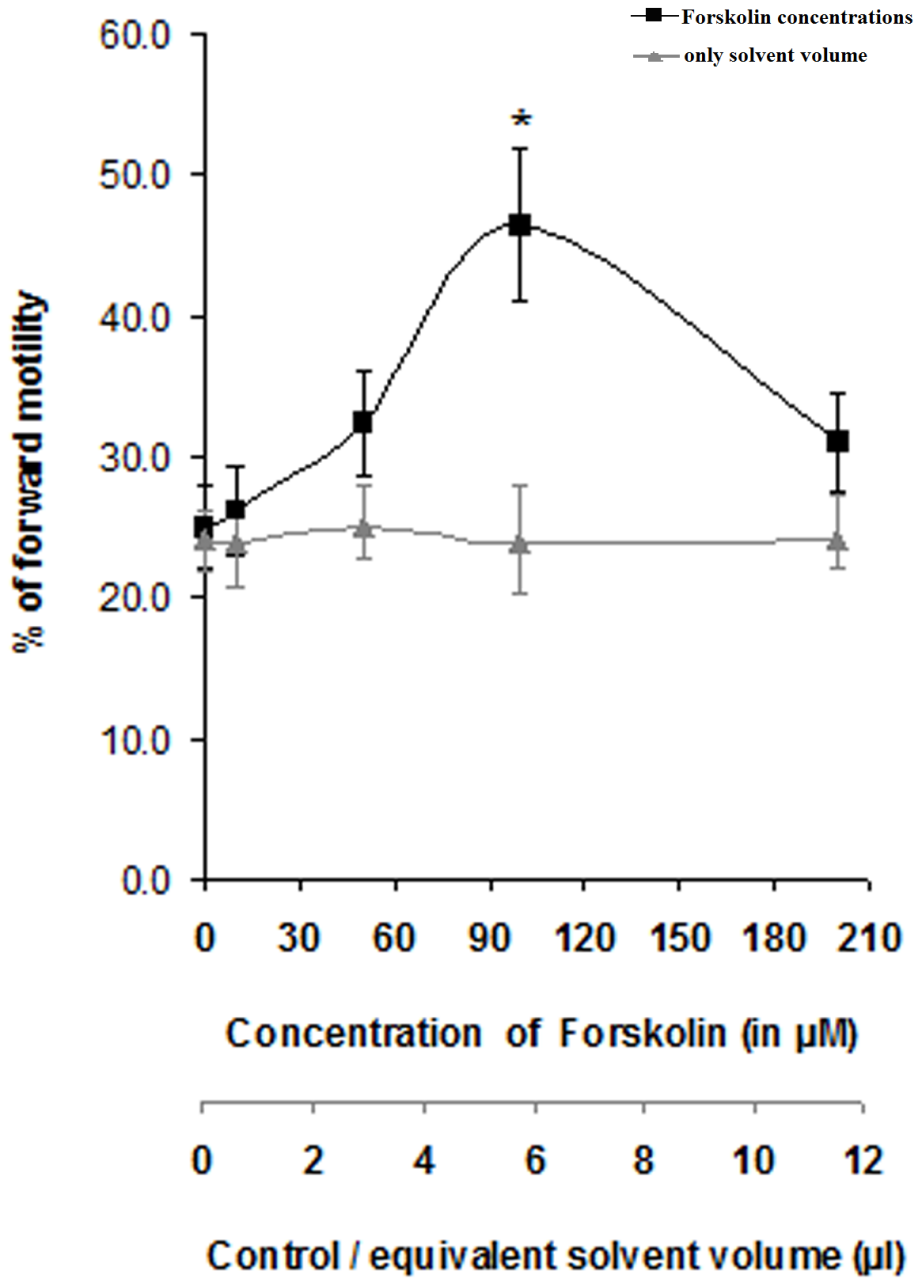
Dose response curve of forskolin on forward motility of mature caprine spermatozoa. Primary X- axis denotes graduations for applied Forskolin concentrations**;** secondary X-axis denotes graduations for control curve where equivalent amounts of solvent of Forskolin that were applied to produce treatment points, were used. The data represent mean ± SEM for n = 3 samples. Asterisk (*) denotes statistically significant difference vs. control (*p<0.05*). Viable cell count throughout the different assay conditions remained ≥97%.

Purified goat sperm membrane was free from any contamination of cytosolic or organelle proteins as judged by the marker enzyme assays and microscopic analysis (data not shown); isolated membrane was found to contain substantial adenylyl cyclase activity under the optimum assay condition. Kinetic properties clearly suggested that its activity is essentially dependent on the presence of Mg^++^ ion (**Table 1**). Forskolin (FSK) at optimum dose was able to increase its activity by around 40% (*p<0.01*), but ddAdo prevented this FSK activity. HCO_3_^−^ or KH7 did not have such altering effect on the adenylyl cyclase activity of the sperm membrane. FSK was also able to considerably augment (*p<0.05*) the number of forward motile cells (**Fig. 2**), signifying the presence of the responsive form of adenylyl cyclase in the goat sperm membrane.

**Table 1:** Study of kinetic properties of adenylyl cyclase activity of purified sperm membrane fraction.

Assay system	Adenylyl cyclase activity	% mean of activity
	(cAMP in pmole/mg/min)	
Complete	37±1.69	100
−Mg^++^	3.8±0.27	10.2
+EDTA (2 mM)	0.94±0.07	2.5
−Mg^++^+Ca^++^ (1 mM)	3.9±0.1	10.5
−Mg^++^+Ni^++^ (0.1 mM)	3.89±0.14	10.5
−ATP	1.15±0.22	3.1
−PCr	31±3.21	83.7
−F^−^	35.9±2.23	97
ddAdo (225 µM)	9.1±0.8	24.6
+Forskolin (0.1 mM)	56.4±0.8	152.4
+ ddAdo+Forskolin	12.4±1.77	33.5
KH7 (25 µM)	35.4±1.57	95.6
+HCO_3_^−^ (50 mM)	35.9±2.25	97

‘Complete’ assay medium for membrane protein AC-activity contains the following: 50 mM Tris-Cl, pH 8.5, 2 mM MgCl_2,_ 0.5 mM NaF, 0.5 mM phosphocreatinine, 1.5 mM ATP-Na_2._ Results represent mean ± SEM for n = 5 experiments.

### Involvement of transmembrane adenylyl cyclase (tmAC) in FMSF induced forward motility

**Figure 3:**
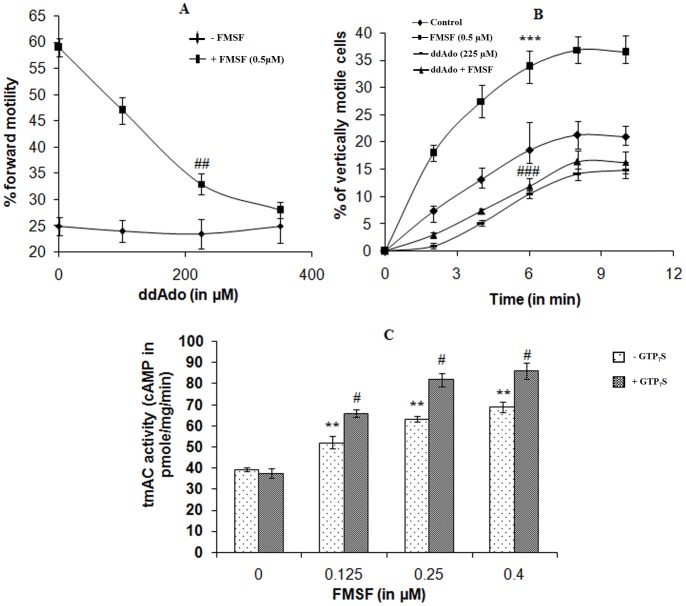
Role of transmembrane adenylyl cyclase in mediation of FMSF activity. **A.** Dose-response curve of ddAdo on effect on forward motility of caudal spermatozoa; **B.** Results of Spectrophotometric vertical motility assay of caprine mature spermatozoa. Viable cell count throughout the different assay conditions remained ≥95%. **C.** Dose-dependency of tmAC activity in presence of FMSF (light bars). Involvement of G_α_-subunit in manifestation of FMSF activity represented by dense bars where GTP_γ_S was used at a concentration of 10 µM. Pre-treatment periods for both ddAdo and GTP_γ_S were of 30 min. Data for figure A and B represent mean ± SEM for n = 5 samples, while that of figure C is of n = 3 samples. Asterisks (***) and hash marks (#, ##, ###) denote statistically significant difference vs. control (*p<0.001*) and vs. only FMSF-treated (*p<0.05, p<0.01 and p<0.001*), respectively.

Inhibition of tmAC with increasing concentrations of ddAdo virtually had no effects on the microscopic forward motility of untreated mature spermatozoa **(** **Fig. 3A** **)**; however the velocity of the treated sperm cells was altered as judged spectrophotometrically **(** **Fig. 3B** **)**. On the other hand, successive doses of the pharmacological blocker of tmAC was able to cut down FMSF-induced progressive motility considerably (*p<0.01*). Moreover, adenylyl cyclase assay with the isolated sperm membrane in presence of increasing concentrations of the glycoprotein factor exhibited a rising trend of activity of the enzyme resulting in boosted generation (*p<0.01*) of cAMP **(** **Fig. 3C** **)**. The data clearly points towards the mediation of adenylyl cyclase in FMSF activity.

### Association of G protein in FMSF induced signaling

**Figure 3C** also reveals the involvement of the G_α_ subunit in the manifestation of the sperm-FMSF interaction. GTP_γ_S, the nonhydrolysable analogue of GTP was found to enhance the FMSF-induced tmAC activity by approximately 25–30% (*p<0.01*) at a concentration of 10 µM. Upto 0.4 µM dose of FMSF, a continuous rise was attained in tmAC activity in presence of GTPγS in the single time-point (30 min) assay.

### Effect of FMSF on regulation of intracellular cAMP level

**Figure 4:**
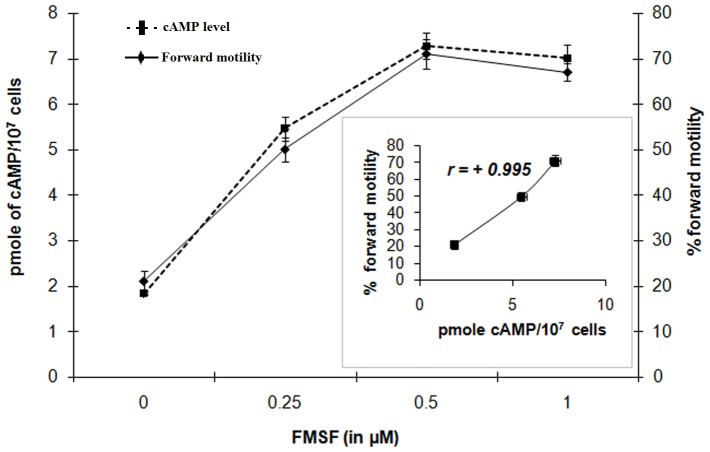
Involvement of cAMP in FMSF-initiated signaling. Dose-response curve of FMSF for generation of intracellular cAMP and corresponding forward motility. **Inset figure** represents correlation between [cAMP]_i_ and forward motility induced by FMSF (*r = +0.995*).

A dose-dependent linear increase in intracellular cAMP level ([cAMP]_i_) was observed with FMSF (*p<0.001*). The curve in **figure 4** clearly demonstrates that the optimal dose (0.5 µM) of FMSF caused more than 3-fold elevation in [cAMP]_i_ and above that point a plateau state was attained. Moreover, this rise in [cAMP]_i_ was in tandem with the percentage of forward motile cells under the influence of FMSF as found in the nearly linear nature of the curve in **inset** **figure 4** **.** The correlation coefficient (*r)* value of +0.995 signifies positive correlation between progressive motility and cAMP levels.

**Figure 5:**
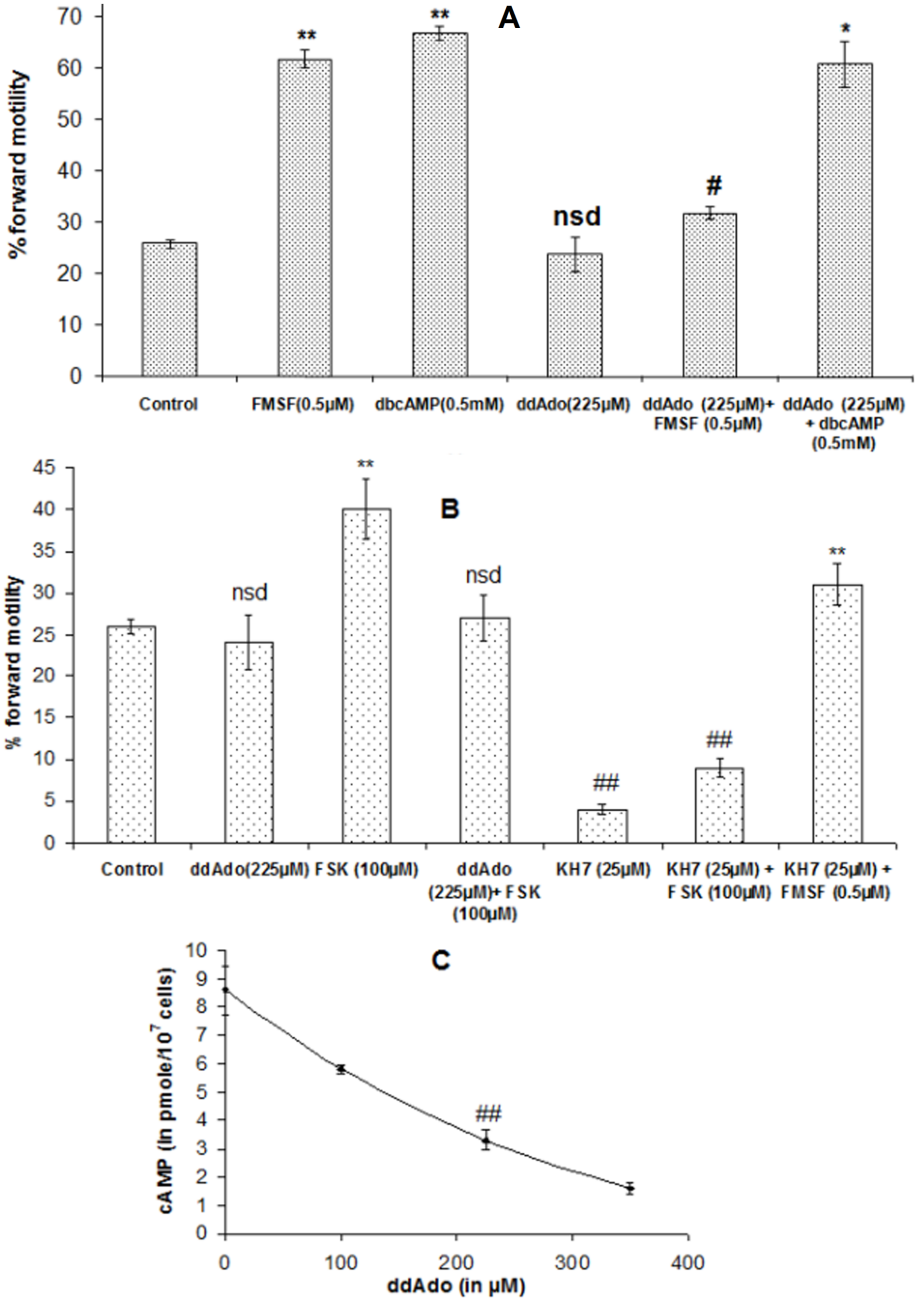
Elucidation of role of tmAC in quality of sperm forward movement. **A.** Effect of ddAdo (dideoxyadenosine) and db-cAMP (dibutyryl cAMP) in relation with FMSF-induced forward motility. **B.** Effect of ddAdo and KH7 in relation with FSK induced forward motility. Incubation periods with ddAdo, KH7, FSK and db-cAMP were of 30 min each. All the data represent mean ± SEM for n = 6 samples. Double asterisks (**) denote statistically significant difference vs. control (*p<0.01*), hash mark (#) indicates statistically significant inhibition vs. FMSF-treated (*p<0.01*), double hash mark (##) indicates statistically significant inhibition vs. control (*p<0.01*), asterisk (*) denotes statistically significant difference vs. (ddAdo+FMSF) (*p<0.05*), *nsd* denotes not significant difference vs. control. Viable cell count throughout the different assay conditions remained ≥95%. **C.** Effect of different doses of ddAdo on modulation of intracellular cAMP level in presence of FMSF (0.5 µM). Duration of ddAdo and FMSF incubation were 30 and 2 min, respectively. All the data represent mean ± SEM for n = 4 samples. Hash mark (##) indicates statistically significant inhibition vs. ddAdo-untreated (*p<0.01*).

This involvement of cAMP in mediation of FMSF activity was further confirmed by the application of its cell permeable analogue, db-cAMP. It induced progressive motility not only in untreated spermatozoa (*p<0.01*), but also in presence of ddAdo (*p<0.05*), the tmAC inhibitor **(** **Fig. 5A** **)**; it mimics the FMSF activity. However, when tmAC was blocked with ddAdo, FMSF induced progressive motility was found to be significantly reduced (*p<0.01*). Since db-cAMP is readily cell permeable, its route of action is tmAC independent; its activity was therefore unaltered even in presence of inhibitor of the tmAC. **Figure 5B** indicates that ddAdo was also able to prevent FSK induced forward motility, but not as much as KH7; these observations clearly imply that sAC is the primary driving force in sperm forward motility at basal state. However, FMSF was able to overcome the motility inhibition caused by KH7 implying it to be a stronger tmAC activator than FSK.

### Alteration in the velocity pattern of vertical motility in presence of FMSF and ddAdo

An interesting finding with regard to quality of velocity under the influence of FMSF and ddAdo was made with spectrophotometric vertical movement analysis **(** **Fig. 3B** **)**. After 10 min incubation, FMSF induced almost double the number of spermatozoa to be vertically motile in comparison with the control set (*p<0.001*). However, a striking difference came out in relation to the microscopic assay result for ddAdo treatment. Pre-incubation with ddAdo (225 µM) significantly impaired vertical motility profile of the spermatozoa (*p<0.001*) below basal value, even in presence of optimum dose of FMSF (*p<0.005*); this qualitative change was not found during horizontal movement analysis. Dey et al., (2014) have previously shown that to a certain extent, some native FMSF molecules are bound to caudal sperm cells at basal state; hence this current finding implies that, at basal state ddAdo reduced sperm velocity by inhibiting tmAC that were recruited by native cell bound FMSF molecules.

### Effect of ddAdo on intracellular cAMP status

**Figure 5C** illustrates the gradual inhibitory effect of increasing doses of the adenylyl cyclase P-site blocker on the intracellular cAMP concentration in presence of the motility stimulatory glycoprotein (*p<0.001*). At 350 µM concentration, the inhibitor was found to bring down [cAMP]_i_ almost to the basal level. For vertical velocity, cells require more kinetic energy as the direction is anti gravity and the [cAMP]_i_ is generally regarded as the kinetic source for the cells. Therefore it can be said that in presence of the selective tmAC blocker, native FMSF molecules attached to sperm surface could not exert their full activity on the cells and as a result of this, remarkably diminished percentage of vertically motile spermatozoa was recorded, even after addition of purified FMSF (**Fig. 3B**).

### Contribution of phosphokinases in manifestation of FMSF activity

**Figure 6:**
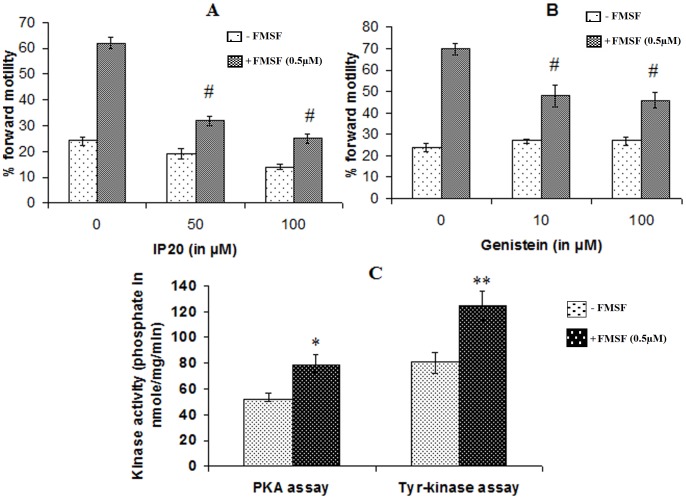
A. Effect of PKA inhibitor in forward motility of the FMSF activated sperm cells. Duration of IP20 incubation was 30 min. B. Effect of tyrosine-kinase inhibitor on forward movement profile of FMSF stimulated spermatozoa. Duration of Genistein incubation was 30 min. Viable cell count throughout the different assay conditions remained ≥95%. **C. Phospho-kinase stimulating potential of FMSF.** Only absorbance values from ELISA results have been plotted, actual activity units have not been shown; anti-phospho-serine/threonine for Protein Kinase A and tyrosine antibodies for Tyrosine kinase were used in ELISA of the assayed treatments. All the data represent mean ± SEM for n = 3 samples. Hash mark (#) indicates statistically significant inhibition vs. only FMSF-treated (*p<0.01*), Asterisks (*, **) denote statistically significant difference vs. control (*p<0.05 and p<0.01*), respectively.

**Figure 7:**
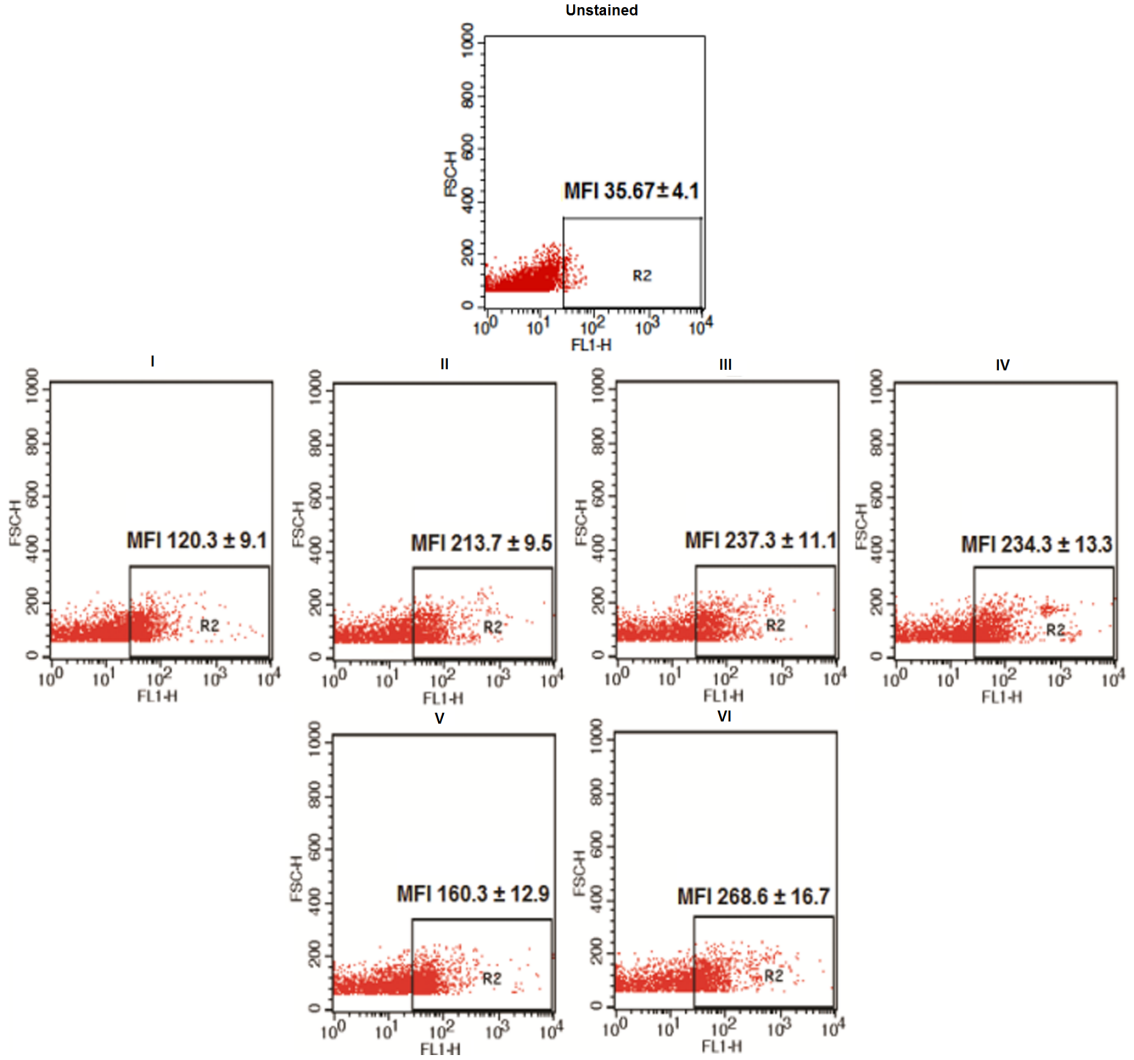
Flow-cytometric analysis of the phosphorylation status of spermatozoa. **Panel I–IV:** Ser/Thr phosphorylation status; **I**- Control, **II-** db-cAMP (0.5 mM), **III**- FMSF (0.5 µM), **IV**- Genistein (250 µM). **Panel V–VI**: Tyr phosphorylation status; **V**- Control, **VI**- FMSF (0.5 µM). The figure is representative and the data represent mean ± SEM for n = 3 samples. The R2 region signifies fluorescence of stained cells (R1, not indicated denotes the entire region); percentage in the figure is of gated cells in the R2 region and corresponding Mean Fluorescence Intensity (MFI) are given beside.

Two of the principal phosphokinase classes viz. serine/threonine kinase and tyrosine kinase were found to have roles in mediation of signaling cascade induced by FMSF. IP20, a competitive inhibitor of cAMP dependent protein kinase A caused considerable reduction (*p<0.01*) in the percentage of FMSF-induced progressively motile cells percentage (**Fig. 6A**). Similar downregulating function was associated with genistein (*p<0.01*), the cell permeable tyrosine kinase inhibitor (**Fig. 6B**). Interestingly, neither of the phosphokinase blockers could fully suppress the stimulated level of forward motility. **Figure 6C** demonstrates the phosphokinase stimulating potential of FMSF. FACS data of **figure 7** displays evidences for the augmented intracellular phosphorylated levels of serine/threonine (**panel III**) and tyrosine residues (**panel VI**) in FMSF-activated sperm cells without notable alteration in the fluorescing cell numbers (data on percentage of cell number not shown). A fascinating aspect about the sequence of activation of the two kinases was available from the above data (**panel I–IV**); genistein could not repress the FMSF-induced serine/threonine phosphorylation intensity which implied that activation of PKA possibly took place upstream of the tyrosine kinase. These data also suggest that FMSF increases motility by instrumenting phosphorylation not necessarily in totally new sets of cells, but by intensifying the former (as evident from corresponding MFI values).

### The tyrosine phosphorylation site in spermatozoa under the influence of FMSF

**Figure 8:**
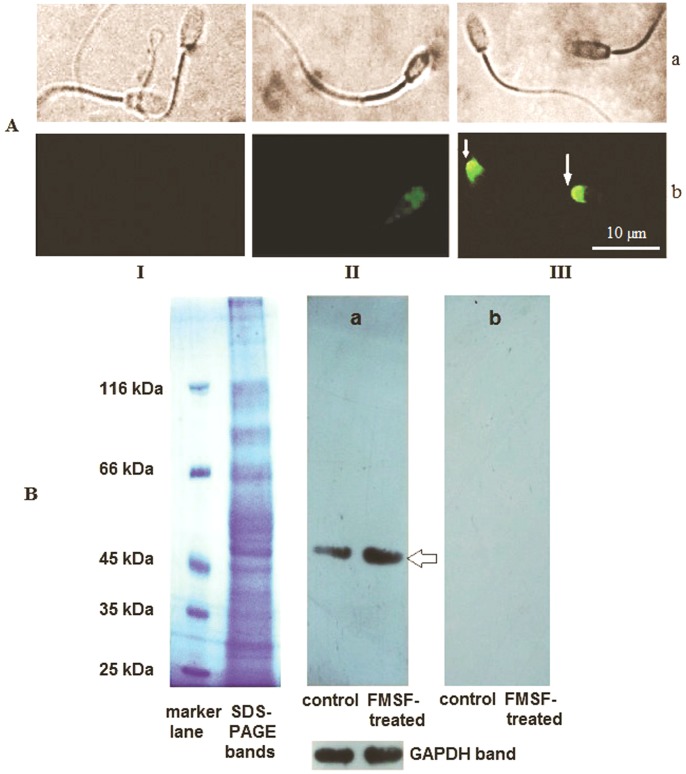
Immuno-localization of Tyrosine-phosphorylation site, (A). **Panel a:** Bright-field images, **Panel b:** Fluorescent images; **I-** Protein A-FITC only control, **II-** untreated, **III**- FMSF (0.5 µM; incubation time 10 min). White arrows indicate sperm head where enhanced phosphorylation was observed. The figure is representative of different such views and was taken at 600× magnification. **B. Western blot profile of tyrosine-phosphorylated protein.** Electrophoresis was done in 10% denaturing polyacrylamide gel. FMSF dose: 0.5 µM/incubation time 10 min. The MW markers used were beta-galactosidase (116 kDa), bovine serum albumin (66.2 kDa), ovalbumin (45.0 kDa), lactate dehydrogenase (35.0 kDa), REase Bsp98I (25 kDa). The set of bands with differential profile has only been shown. The SDS-PAGE band is of whole cell lysate; panels (**a**) and (**b**) correspond to developed Western blot of whole cell lysate protein and membrane protein fraction, respectively. Arrow indicates the detected ≈ 50 kDa band.

Effect of tyrosine kinase in relation to FMSF signaling was found at the anterior portion of the sperm head (**Fig. 8A**). Fluorescent microscopic visuals indicated the sperm anterior head to be the tyrosine-phosphorylated site (**panel bII, III of 8A**). FMSF treated cells displayed considerably greater fluorescence than the untreated ones, signifying enhanced phosphorylation.

### Identification of the tyrosine phosphorylated protein

Only one differentially visible tyrosine phosphorylated protein band was detected by the Western blot analysis of the whole cell lysate protein fraction of the sperm cells **(** **Fig. 8B** **- panel a)**. **Panel b of** **figure 8B** clearly shows that the sperm membrane protein fraction was not detected by this particular anti-phosphotyrosine antibody. The single time-point FMSF treatment resulted in greater phosphorylation of the protein band of ≈ 50 kDa molecular weight. However, the time-dependent phosphorylation status was not checked as FMSF induces motility instantaneously in sperm cells.

## Discussion

We report for the first time a defined mechanism for the regulation of the progressive motility of mammalian spermatozoa by a purified extracellular factor. Enhancement of forward motility of goat sperm cells by FMSF may open a new avenue for the scientific communities to search and purify more such molecules from mammalian body fluids. FMSF is already biochemically a well-characterized molecule [11]. In this present article we have provided MS analysis data to strengthen its characterization as a novel protein identified in biological system. The homology study with the obtained MS data shows a maximum of 37% similarity of FMSF with transferrin or transferrin like glycoproteins. Transferrin or transferrin like proteins has previously been implicated to have some role in sperm function [41], [42]; however, FMSF is clearly different from transferrins in terms of molecular weight, isoelectric pH and some other biochemical properties. Previous publication from our laboratory has provided evidences of immuno-reactive presence of FMSF in reproductive fluids of certain cattle species and human [16], [20]. Its receptor has been identified and linked with epididymal maturation of goat sperm cells. It has been indicated that functionally active receptors of FMSF may be present on sperm surface of other mammals [11], [16], as well. Therefore, the crucial point was to unravel the mechanism of action of this extracellular glycoprotein factor.

Studies in the last one decade have certainly brought forward the novel facet of presence of functional transmembrane adenylyl cyclase in sperm cells. But, activation of this adenylyl cyclase by any form of physiological factor or ligand for intonation of sperm progressive motility was unreported. This article identifies the so called illusive molecule, which upon binding to the sperm surface receptor activates tmAC to generate cAMP and promotes forward movement, as the forward motility stimulating factor (FMSF). Thus, this glycoprotein factor is unique in its operation. Previously, the pattern of progressive or forward motility of spermatozoa was attributed only to its distinct soluble adenylyl cyclase (sAC) activity [43], [44], [45]. But, our finding for the first time indisputably suggests that transmembrane adenylyl cyclase is involved in stimulating forward motility recruited by an extracellular physiologic factor, viz. FMSF. It showed strong forward motility inducing activity even when sperm cells lacked the contribution of sAC. This very finding denotes a distinctive phenomenon exhibited by any physiologic molecule. However, any downstream effect of FMSF binding to sperm cells, on sAC activity could not be inferred from this study. Since KH7 is a long lasting inhibitor of sAC, FMSF could not possibly have stimulated the latter in presence of KH7 in such short span of incubation. But, it does not necessarily rule out the possibility of any indirect modulation of sAC by FMSF in uninhibited i.e. normal state. The obtained set of data also proves that FMSF is more effective stimulator of tmAC than Forskolin. There are indirect reports from previous researchers which supports this notion of involvement of tmAC in regulation of sperm motility; marked reduction of swimming ability of murine spermatozoa as a consequence of disruption of AC3 gene, a ubiquitous eukaryotic tmAC was reported a few years ago [46]. There are also information on anticipated participation of transmembrane adenylyl cyclase in regulation of forward motility in bovine, human and amphibian spermatozoa [47], [48], [49]. Hence, efforts for the isolation of similar motility enhancing physiologic molecules in other animal systems and elucidation of their mechanism of action may be boosted in the coming years by our findings.

Regulation and contribution of different phosphokinases in sperm forward motility at basal level is not well understood till date. The present study also illuminates on this aspect to demonstrate a potential crosstalk between the two phosphokinases in manifestation of FMSF stimulated forward motility. Interestingly enough, cAMP dependent protein kinase A and intracellular tyrosine kinase were found to be partially responsible for stimulation FMSF induced motility. However studies with optimal doses of their selective inhibitor showed that for maximal response of effect of FMSF these phosphokinases are absolutely essential. Dependence of sperm motility on PKA has been shown in the past, as well [50]. Sequence analysis of their activation profile suggests that cAMP-dependent PKA is activated upstream of the tyrosine-kinase (Fig. 7). It may easily be assumed that increased the [cAMP]_i_ pool might have activated the PKA and tyrosine-kinase may have been turned on later. But, whether PKA directly activates the latter, remains unresolved and require further investigation. The detected ≈50 kDa tyrosine-phosphorylated protein identified has resemblance with a previously reported protein in capacitating conditions of goat sperm [51]. In both the cases, tyrosine phosphorylation site have been found on sperm head. Similar observations regarding site of tyrosine phosphorylation in spermatozoa have been reported for human and boar system, too [52], [53]. We postulate that the signaling cascade which originates with FMSF-receptor interaction and leads to tyrosine phosphorylation on sperm head may induce other intracellular molecular entities to transmit the signal to manifest movement of sperm tail. However, the exact downstream functions of these intracellular phosphokinases and for that matter other possible role of the enhanced interior cAMP pool could not be delineated from the present observations. The ultimate materialization of FMSF initiated signaling can only be explored if perceptions of the preceding events are clear. Plausible involvement of other regulators of cellular machinery that had long been associated with sperm motility [1] needs to be inspected under the influence of FMSF. In this regard, the roles of calcium ion, calmodulin, cytosolic adenylyl cyclase and cAMP directed phosphodiesterases and protein kinase A anchoring proteins require further studies; the physiological significance of this enhanced forward motility remains to be checked in animal model. But, the *in vivo* presence of FMSF as a part of the protein repertoire of the reproductive fluids and its defined signaling mechanism has already implicated it to be a potentially significant biomolecule in the field of reproductive biology.
